# Large mid-upper arm circumference is associated with reduced insulin resistance independent of BMI and waist circumference: A cross-sectional study in the Chinese population

**DOI:** 10.3389/fendo.2022.1054671

**Published:** 2022-12-23

**Authors:** Jialu Wang, Liyun He, Na Yang, Ziyi Li, Lingling Xu, Wei Li, Fan Ping, Huabing Zhang, Yuxiu Li

**Affiliations:** Department of Endocrinology, Key Laboratory of Endocrinology of National Health Commission, Peking Union Medical College Hospital, Chinese Academy of Medical Sciences & Peking Union Medical College, Beijing, China

**Keywords:** mid-upper arm circumference, insulin resistance, mid-arm muscle circumference, triceps skinfold thickness, body composition

## Abstract

**Background:**

Body mass index (BMI) is a common indicator in clinical practice, but it is not sufficient to predict insulin resistance (IR). Other anthropometric methods supplement BMI in the assessment of body composition, which can be predicted more accurately. This cross-sectional study aimed to evaluate the association between mid-upper arm circumference (MUAC), triceps skinfold (TSF) thickness, mid-arm muscle circumference (MAMC) and IR in Chinese adults.

**Methods:**

This cross-sectional study analyzed data from the 2009 China Health and Nutrition Survey database. The study population was divided into four groups according to the MUAC quartiles, and the homeostasis mode assessment was used to evaluate the degree of IR. Logistic regression analysis was performed to calculate odds ratios (ORs) with 95% confidence intervals (CIs), with adjustments for multiple covariates. Subgroup analyses stratified by age, sex, BMI, waist circumference (WC), smoking status, and alcohol consumption were performed.

**Results:**

In total, 8,070 participants were included in the analysis. As MUAC increased, BMI, TSF thickness, MAMC, and the proportion of IR tended to increase. However, we found that there was a significant negative association between MUAC and MAMC and IR in the logistic regression analysis, independent of BMI and WC, the ORs for the highest quartiles compared with the lowest quartiles were 0.662 (95%CI: 0.540-0.811) and 0.723 (95%CI: 0.609-0.860), respectively. There was no significant association was observed between the TSF thickness and IR (OR=1.035 [95%CI: 0.870-1.231]). The inverse associations were more pronounced among participants with lower BMI and WC. No significant age-specific differences were observed (P-heterogeneity > 0.05).

**Conclusions:**

After adjusting for BMI and WC, MUAC was negatively associated with IR in Chinese adults, and the association between MUAC and IR was derived from arm muscle instead of subcutaneous fat. MUAC could be an additional predictor of IR besides BMI and WC in clinical practice.

## 1 Introduction

Insulin resistance (IR) plays an important role in the development of metabolic syndrome and type 2 diabetes mellitus (T2DM) (1, 2). The incidence of metabolic syndrome and T2DM is increasing, and T2DM has become a major burden on the healthcare system worldwide, especially in China (3, 4). In addition, IR and metabolic disorders are positively associated with all-cause mortality (5, 6). Therefore, early detection of IR and intensive intervention are effective ways to reduce metabolic diseases and mortality.

Many studies have suggested that anthropometric measurements, such as body mass index (BMI) and waist circumference (WC), can be used as indicators of IR (5, 7–11). However, BMI can only be used as an indicator of overall obesity, and WC is associated with visceral fat (12, 13). Muscle and subcutaneous fat also play a role in the development of IR. Therefore, identifying indicators that can represent muscle and subcutaneous fat and predict IR is important.

Mid-upper arm circumference (MUAC) is a readily available, simple, and inexpensive indicator (14), and some studies have proposed that it could replace other anthropometric measurements as a new indicator for predicting IR. However, different results have been reported regarding the relationship between MUAC and IR. Most studies indicated a positive association between MUAC and the degree of IR (12, 15–17), whereas some studies did not observe this correlation (5). The participants in most studies were mostly middle-aged and elderly adults (5, 16, 17). Several studies were limited to specific populations, such as those with T2DM and obesity (12, 15). In addition, MUAC consists of mid-upper arm fat, which is indicated by triceps skinfold (TSF) thickness, and mid-upper arm muscle, which is indicated by mid-arm muscle circumference (MAMC) (18, 19). Different components may have diverse mechanisms in the metabolic process. To the best of our knowledge, few studies have evaluated the relationship between mid-arm measurements and IR in Chinese adults and examined the effects of muscle and subcutaneous fat. This study aimed to explore the association between MUAC and IR in the Chinese adult population using the China Health and Nutrition Survey (CHNS) database and the roles of TSF thickness and MAMC in the relationship.

## 2 Materials and methods

### 2.1 Study population

The CHNS is an ongoing open large-scale cohort study in China. The CHNS comprised ten rounds of surveys between 1989 and 2015 for investigating the impact of social and economic transformation on the health and nutritional status of the Chinese population. A multistage randomized cluster sampling method was used to select samples from both rural and urban areas of nine representative provinces in mainland China, covering most of the northern and southern regions (20, 21). Detailed information on the survey design and methodology has been reported previously (22). The study was conducted in collaboration with the University of North Carolina at Chapel Hill and the National Institute of Nutrition and Food Safety of the Chinese Center for Disease Control and Prevention, with CHNS data provided by the website (https://www.cpc.unc.edu/projects/china).

In this study, we used CHNS data from 2009, when blood samples were first collected. A total of 9,549 CHNS participants were enrolled (23). Participants were excluded if they met any of the following criteria: missing age and sex data, age <18 years, missing laboratory or anthropometric data, participants who were pregnancy or breast-feeding, fasting glucose levels <3.5 mmol/L, and participants with extreme MUAC values greater than or less than the mean ± three standard deviations. In addition, participants receiving glucose-lowering therapy were also excluded from analysis because their decreased beta-cell function could potentially result in an inaccurate homeostatic model assessment for insulin resistance (HOMA-IR) value.

All research procedures were conducted in accordance with the tenets of the Declaration of Helsinki (as revised in 2013) and were approved by the institutional review boards of the University of North Carolina at Chapel Hill, the National Institute for Nutrition and Health, and the Chinese Center for Disease Control and Prevention. Consent was obtained from each participant.

### 2.2 Mid-arm measurements

Mid-arm measurements were performed by trained investigators following the anthropometric standards recommended by the World Health Organization (20). Three measurements were taken for each participant and the mean of these measurements was used in the analysis. With the participant’s elbow fully extended, the MUAC was measured at the midpoint between the ulnar eminence and the acromion of the scapula with an accuracy of 0.1 cm (24). TSF thickness was measured at the midpoint of the posterior line between the olecranon and tip of the acromion using a skinfold caliper and recorded to the nearest 0.5 mm (24). The muscle circumference of the mid-upper arm was calculated using a standard formula (19).

### 2.3 Data collection of covariates

Participants wore light clothing, and their weight was measured using a calibrated beam scale with a weight measurement accurate to 0.1 kg. The height of the participants without shoes was measured using a portable stadiometer accurate to 0.1 cm. BMI was calculated as weight (kg) divided by height (meters) squared. WC was measured at the midpoint between the lowest rib and iliac crest using a non-elastic tape (20). According to the modified NCEP criteria, the cut-off points for WC should be ethnic-specific. Central obesity defined as WC ≥ 90 cm in men or ≥ 85 cm in women in Chinese population (25). Blood pressure was measured three times with the participant in a seated position at 10 min intervals, and the average values of the systolic blood pressure (SBP) and diastolic blood pressure (DBP) were calculated.

A standardized questionnaire was used by the staff to collect background information, medical history, and lifestyle information, including age, sex, educational attainments(low: lower middle school or below; medium: higher middle school or vocational/technical school; high: college/university or higher), smoking status (current/ever smoking or not), alcohol consumption (current/ever drinking or not), and physical activity (low, medium, high) (26). Blood samples were collected from all participants after 12–14 hours of fasting and were stored in test tubes. All blood samples were analyzed at the central laboratory of the China–Japan Friendship Hospital. Fasting blood glucose was measured with a glucose oxidase–peroxidase kit (Landau, UK) (27). The total energy intake was obtained from three consecutive day recalls at the individual level in combination with a weighed food record at the household level (22). Further details on the data are available at https://www.cpc.unc.edu/projects/china. IR was measured using the HOMA-IR as described by Matthews et al. (28). The formula for calculating HOMA-IR was HOMA-IR (mmol/L^2^) = fasting insulin (mmol/L) × fasting glucose (mmol/L)/22.5. IR was defined as the upper quartile of the HOMA-IR values.

### 2.4 Statistical analysis

Statistical analysis was performed using the SAS 9.4 statistical software (version 9.4; SAS Institute, Cary, North, USA). Continuous variables were expressed as mean ± standard deviation (M ± SD), and categorical data were expressed as percentages or frequencies. The participants were divided into four groups according to the MUAC quartiles. ANOVA test was performed to compare between-group differences for continuous data, and the Chi-squared test was used to compare between-group differences for categorical data. To investigate whether the effect of MUAC on IR was mainly due to TSF thickness or MAMC, logistic regression was used to evaluate the relationship between MUAC, MAMC, and TSF thickness and IR, respectively. Possible confounding factors were adjusted in the regression models. Model 1 was not adjusted for confounding factors. Model 2 was adjusted for age, sex, smoking, alcohol consumption, physical activity, energy intake, and education level. Model 3 was adjusted for the variables in Model 2 plus total cholesterol (TC), triglycerides (TG), SBP, and DBP. Model 4 was adjusted for the variables in Model 3 and BMI. Model 5 was further adjusted for WC based on Model 4 to eliminate the effect of visceral fat. Subgroup analysis stratified by age, sex, BMI, WC, smoking status, and alcohol consumption was performed to explore the potential effect modification. A two-sided test with P < 0.05 indicates a significant difference.

## 3 Results

### 3.1 Baseline characteristics of the participants

**Figure 1:**
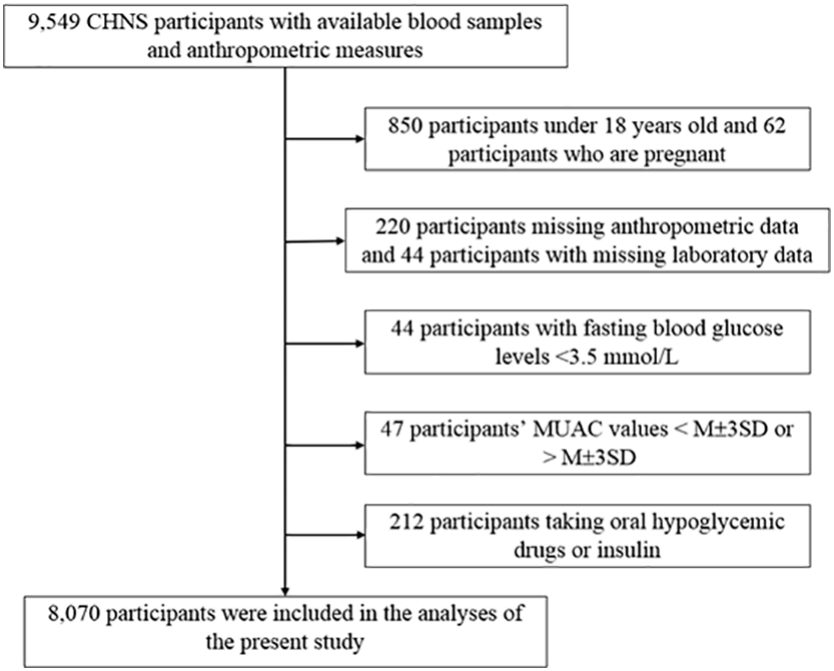
Flowchart of study participants. CHNS, the China Health and Nutrition Survey; MUAC, mid-upper arm circumference; M, mean; SD, Standard deviation.

After applying the exclusion criteria, 8,070 participants with a mean age of 50.2 years, including 4,301 women (53.3%) and 3,769 men (46.7%), were finally included in the study (**Figure 1**). The numbers of participants younger than 40, 40-60, and older than 60 were 2019 (25%), 3853 (48%), and 2198 (27%), respectively. The average MUAC, MAMC, and TSF thickness in the whole population were 27.1 (3.6) cm, 21.9 (3.4) cm, and 16.4 (7.7) mm, respectively. The study population was divided into four groups according to the MUAC quartiles. As the MUAC levels increased, the proportion of men, smoking, drinking, IR, and central obesity tended to increase. Participants in the highest MUAC quartile demonstrated high values in height, weight, BMI, TSF thickness, MAMC, hip circumference, WC, SBP, DBP, fasting blood glucose, fasting insulin, HbA1c, TG, TC, HOMA-IR, low-density lipoprotein cholesterol, and C-reactive protein levels; however, the participants in the highest MUAC quartile exhibited low high-density lipoprotein cholesterol levels. There were no statistical differences in educational attainment levels among the groups (**Table 1**).

**Table 1:** Baseline characteristics of the study population according to mid-upper arm circumference quartiles.

Characteristics	MUAC (cm)				P value
	Q1 (13.0-25.0)	Q2 (25.1-27.0)	Q3 (27.1-29.3)	Q4 (29.4-41.5)	
n	2016	2018	2017	2019	NA
Age (years)	52.23 ± 17.92	49.74 ± 14.94	50.15 ± 13.55	49.78 ± 12.76	<0.001
Male (n, %)	744 (39.90)	914 (45.29)	966 (47.89)	1145 (56.71)	<0.001
Educational attainment level (n, %)					0.1091
Low	1577 (78.22)	1544 (76.51)	1546 (76.65)	1505 (74.54)	
Medium	341 (16.91)	386 (19.13)	381 (18.89)	404 (20.01)	
High	98 (4.86)	88 (4.36)	90 (4.46)	110 (5.45)	
Smoking (n, %)	554 (26.98)	602 (29.83)	645 (31.98)	718 (35.56)	<0.001
Alcohol (n, %)	499 (24.21)	643 (31.86)	681 (33.76)	848 (42.00)	<0.001
Height (cm)	157.86 ± 8.61	160.38 ± 8.22	161.57 ± 7.98	164.30 ± 8.28	<0.001
Weight (kg)	51.31 ± 8.43	57.17 ± 7.45	62.44 ± 7.46	71.66 ± 9.87	<0.001
BMI (kg/m^2^)	20.59 ± 2.56	22.22 ± 2.37	23.92 ± 2.37	26.55 ± 3.11	<0.001
MUAC (cm)	22.60 ± 1.93	26.00 ± 0.70	28.14 ± 0.68	31.61 ± 1.97	<0.001
TSF thickness (mm)	12.72 ± 6.32	14.95 ± 6.76	17.27 ± 7.16	20.72 ± 7.98	<0.001
MAMC (cm)	18.60 ± 2.66	21.30 ± 2.15	22.72 ± 2.28	25.11 ± 2.90	<0.001
Hip circumference (cm)	88.50 ± 6.47	92.17 ± 5.80	95.78 ± 5.99	100.98 ± 6.83	<0.001
WC (cm)	74.96 ± 8.25	79.49 ± 8.12	84.29 ± 7.74	91.27 ± 8.67	<0.001
SBP (mmHg)	121.58 ± 19.19	122.92 ± 18.55	125.20 ± 18.57	128.71 ± 18.39	<0.001
DBP (mmHg)	79.54 ± 13.68	81.62 ± 13.27	83.15 ± 13.44	86.77 ± 14.06	<0.001
FBG (mmol/L)	5.23 ± 1.17	5.26 ± 1.26	5.34 ± 1.17	5.49 ± 1.41	<0.001
FINS (uIU/mL)	12.59 ± 22.75	13.53 ± 26.88	14.14 ± 19.07	16.22 ± 18.33	<0.001
HbA1c (mmol/L)	5.48 ± 0.90	5.51 ± 0.74	5.63 ± 0.81	5.71 ± 0.80	<0.001
TG (mmol/L)	1.37 ± 1.19	1.50 ± 1.21	1.67 ± 1.35	2.08 ± 1.87	<0.001
HDL_C (mmol/L)	1.54 ± 0.49	1.47 ± 0.46	1.39 ± 0.42	1.32 ± 0.43	<0.001
LDL_C (mmol/L)	2.84 ± 0.97	2.95 ± 0.93	3.01 ± 0.96	3.11 ± 1.02	<0.001
TC (mmol/L)	4.73 ± 0.96	4.81 ± 1.01	4.86 ± 0.99	5.03 ± 1.02	<0.001
HOMA-IR	3.18 ± 7.03	3.35 ± 6.98	3.59 ± 5.83	4.35 ± 7.62	<0.001
CRP (mg/L)	2.92 ± 14.01	1.98 ± 4.07	2.35 ± 6.51	2.79 ± 5.65	0.002
Central obesity	191 (9.47)	379 (18.78)	701 (34.75)	1333 (66.02)	<0.001
Insulin resistance (n, %)	390 (19.35)	414 (20.52)	494 (24.49)	720 (35.66)	<0.001

Data are presented as mean ± SD, or n (%). P values are for any difference across the quartiles of MUAC using ANOVA or χ2test as appropriate.

BMI, body mass index; WC, waist circumference; MUAC, mid-upper arm circumference; TSF thickness, triceps skinfold thickness; MAMC, mid-arm muscle circumference; SBP, systolic blood pressure; DBP, diastolic blood pressure; FBG, fast blood glucose; FINS, fasting insulin; TG, triglyceride; TC, total cholesterol; HDL_C, high-density lipoprotein cholesterol; LDL_C, low-density lipoprotein cholesterol. NA, not applicable.

### 3.2 Association of MUAC, MAMC, and TSF thickness with IR

MUAC, TSF thickness, and MAMC were divided into quartiles, with the lowest quartile considered as the reference group. Logistic regression analysis was performed to evaluate the association between MUAC, MAMC, and TSF thickness and IR after adjusting for covariates. In Models 1–3, MUAC, MAMC, and TSF thickness were significantly positively associated with IR (P < 0.001) (**Table 2**). However, after further adjustment for BMI, the relationship between MUAC and IR reversed. MUAC was negatively associated with IR, and the odds ratio (OR) was 0.763 (95% confidence interval [CI]: 0.627–0.929) for the highest versus the lowest quartiles. Similar to MUAC, MAMC became a protective factor in Model 4, and the OR was 0.756 (95%CI: 0.637–0.897) for the highest quartile compared to the lowest quartile. Unlike MUAC and MAMC, after further adjustment for BMI in Model 4, the relationship between TSF thickness and IR was insignificant, and the OR for the highest quartile was 1.117 (95%CI: 0.942–1.326) compared to the lowest quartile.

**Table 2:** Association between mid-upper measurements and insulin resistance in participants.

	Model 1	Model 2	Model 3	Model 4	Model 5
MUAC					
Q1 (lowest)	Reference	Reference	Reference	Reference	Reference
Q2	1.076 (0.922-1.256)	1.127 (0.964-1.317)	1.041 (0.887-1.222)	0.806 (0.683-0.952)	0.775 (0.656-0.916)
Q3	1.352 (1.164-1.571)	1.408 (1.210-1.639)	1.225 (1.047-1.433)	0.741 (0.624-0.880)	0.682 (0.572-0.812)
Q4 (highest)	2.311 (2.003-2.667)	2.445 (2.109-2.834)	1.861 (1.593-2.175)	0.763 (0.627-0.929)	0.662 (0.540-0.811)
P value	<0.001	<0.001	<0.001	0.006	<0.001
MAMC					
Q1 (lowest)	Reference	Reference	Reference	Reference	Reference
Q2	0.982 (0.848-1.137)	0.992 (0.856-1.150)	0.954 (0.820-1.111)	0.836 (0.715-0.976)	0.828 (0.708-0.969)
Q3	1.011 (0.873-1.169)	1.059 (0.912-1.231)	0.965 (0.827-1.127)	0.737 (0.628-0.866)	0.718 (0.611-0.844)
Q4 (highest)	1.467 (1.275-1.687)	1.597 (1.375-1.854)	1.300 (1.112-1.519)	0.756 (0.637-0.897)	0.723 (0.609-0.860)
P value	<0.001	<0.001	<0.001	0.0013	<0.001
TSF Thickness					
Q1 (lowest)	Reference	Reference	Reference	Reference	Reference
Q2	1.229 (1.056-1.432)	1.239 (1.060-1.449)	1.113 (0.947-1.307)	0.937 (0.795-1.105)	0.898 (0.760-1.060)
Q3	1.486 (1.281-1.725)	1.521 (1.302-1.777)	1.318 (1.122-1.548)	1.005 (0.851-1.188)	0.949 (0.801-1.124)
Q4 (highest)	2.034 (1.760-2.351)	2.109 (1.811-2.455)	1.774 (1.515-2.077)	1.117 (0.942-1.326)	1.035 (0.870-1.231)
P value	<0.001	<0.001	<0.001	0.1613	0.2817

Data are presented as coefficients (95% CI). Insulin resistance was defined by the upper quartile of HOMA-IR. All models were constructed using the logistic regression analysis. Model 1 was not adjusted for any confounders; Model 2 was adjusted for age, sex, smoking, alcohol consumption, physical activity, energy intake, and education level; Model 3 was adjusted for the variables in Model 2 plus total cholesterol, triglyceride, systolic blood pressure, and diastolic blood pressure; Model 4 was adjusted for the variables in Model 3 plus BMI. Model 5 was adjusted for the variables in Model 4 plus waist circumference.

BMI, body mass index; MUAC, mid-upper arm circumference; MAMC, mid-arm muscle circumference; TSF thickness, triceps skinfold thickness.

With further adjustment for WC, MUAC and MAMC consistently remained inversely associated with IR, with the effect being more pronounced (**Table 2**), and the ORs of the highest quartiles over the lowest quartiles were 0.662 (95%CI: 0.540–0.811) and 0.723 (95%CI: 0.609-0.860), respectively. TSF thickness still did not indicate an association with IR with further adjustment for WC, and the OR for the highest quartile was 1.035 (95%CI: 0.870-1.231) over the lowest quartile.

### 3.3 Subgroup analyses stratified by age, sex, BMI, WC, smoking status, and alcohol consumption

Subgroup analyses were conducted to explore whether the relationships between MUAC, MAMC, and TSF thickness and IR were influenced by other potential factors (**Table 3**). The models were adjusted for age, sex, smoking status, alcohol consumption, physical activity, educational attainment levels, energy intake, TC, TG, SBP, DBP, BMI, and WC. The modified effects of BMI and WC on the relationship between MUAC and IR were significant (P-heterogeneity < 0.05). The protective effect of MUAC on IR attenuated as BMI and WC increased. We observer a declining trend in the association between MAMC and IR with increasing BMI or WC, which was mainly consistent with MUAC. No significant modified effects of age, sex, smoking status or alcohol consumption were observed (P-heterogeneity > 0.05).

**Table 3:** Association between mid-upper measurements and insulin resistance stratified by age, sex, BMI, WC, smoking status, and alcohol consumption.

	MUAC		MAMC		TSF thickness	
	OR (95% CI)	P - interaction	OR (95% CI)	P - interaction	OR (95% CI)	P - interaction
Age		0.7728		0.8815		0.5908
Age <40	0.705 (0.461, 1.078)		0.698 (0.486, 1.003)		1.017 (0.701, 1.475)	
40≤ Age <60	0.585 (0.429, 0.797)		0.683 (0.530, 0.879)		1.007 (0.788, 1.313)	
Age ≥60	0.917 (0.636, 1.321)		0.855 (0.619, 1.182)		1.076 (0.782, 1.481)	
Sex		0.6711		0.9153		0.9884
Male	0.543 (0.400, 0.737)		0.681 (0.528, 0.878)		0.989 (0.774, 1.265)	
Female	0.802 (0.609,1.057)		0.799 (0.621, 1.027)		0.994 (0.770, 1.283)	
BMI		<0.001		0.0051		0.0023
BMI < 20	0.613 (0.168, 2.236)		0.342 (0.132, 0.885)		0.840 (0.433, 1.627)	
20 ≤ BMI ≤ 24	0.702 (0.496, 0.994)		0.677 (0.492, 0.932)		0.972 (0.740, 1.278)	
BMI > 24	0.788 (0.559, 1.111)		0.726 (0.544, 0.969)		1.271 (0.968, 1.669)	
WC		0.0033		0.0022		0.0210
Non central obesity	0.617 (0.468-0.812)		0.609 (0.480-0.773)		1.094 (0.871-1.374)	
Central obesity	0.835 (0.570-1.223)		0.911 (0.690-1.201)		0.998 (0.743-1.340)	
Smoking status		0.0628		0.0542		0.6571
Ever/current smoker	0.504 (0.344, 0.737)		0.559 (0.413, 0.757)		1.150 (0.846, 1.564)	
Never smoker	0.748 (0.588, 0.951)		0.803 (0.649, 0.992)		0.962 (0.778, 1.191)	
Alcohol consumption		0.3189		0.2585		0.6024
Drinker	0.474 (0.328, 0.685)		0.589 (0.436, 0.797)		1.205 (0.893, 1.627)	
Non-drinker	0.779 (0.609, 0.995)		0.811 (0.654, 1.004)		0.940 (0.759, 1.164)	

All models were adjusted for age, sex (male or female), smoking status (ever/current or never smoker), alcohol consumption (yes or no), physical activity (low, medium, or high), educational attainment levels (low, medium, or high), energy intake, total cholesterol, triglyceride, systolic blood pressure, diastolic blood pressure, BMI, and waist circumference. The results were the OR (95% CI) of insulin resistance calculated for the highest quartile of MUAC, MAMC, and TSF thickness compared to the lowest quartile.

OR, odds ratio; CI, confidence index; BMI, body mass index; WC, waist circumference, MUAC, mid-upper arm circumference; MAMC, mid-arm muscle circumference; TSF thickness, triceps skinfold thickness.

## 4 Discussion

In this study, we observed a negative association between MUAC and MAMC and IR, which is independent of BMI and WC. No significant association was observed between TSF thickness and IR. The results of MUAC and MAMC were consistent, which may indicate that the protective effect on IR mainly comes from muscle but not subcutaneous fat. In addition, the finding did not differ significantly among different age groups. The results implied that MUAC could be used to predict IR and the effects of MUAC on IR should receive more attention.

In clinical practice and research, BMI representing overall obesity is considered to be associated with metabolic risk and can predict IR. But substantial evidence has demonstrated that visceral fat has a detrimental effect on IR. Therefore, WC is often used as an indicator of visceral fat mass to help further screen for individuals with high metabolic risk (12, 13). Furthermore, whether other anthropometric methods independent of BMI and WC can help better predict metabolic risk. As a readily available, simple, inexpensive, and non-invasive anthropometric measurement, MUAC is often used as an indicator to assess nutritional status (29, 30). However, many recent studies have explored the association between MUAC and metabolic risk factors (15, 16). Several studies have revealed the association between MUAC and IR, some of which have identified a positive association between MUAC and IR (5, 12). But these studies did not adjust for the effect of BMI on the relationship between MUAC and IR, which may not reflect the true relationship. Only one study has reported that the association between MUAC and IR disappeared after adjusting for BMI in logistic regression model (5). The study population was from the United States, whereas the study population in the current study was from China, ethnic differences may have contributed to the different results. Their study focused on middle-aged and elderly adults, while the current study population comprised adults aged >18 years.

Furthermore, the relationship between MAMC and IR was consistent with that between MUAC and IR, which may indicate that the protective effect of MUAC on IR is derived from MAMC. Similar results have been reported in previous studies, with consistent effects of MUAC and MAMC on clinical outcomes (31–33). In our study, MUAC and MAMC levels were negatively associated with IR. However, different results have also been reported. A previous study discovered that greater muscle mass may promote the development of IR (8, 34); but it did not adjust for BMI as a confounding factor, which may account for the different results. By contrast, the findings of a cross-sectional study were consistent with the present study that IR was significantly associated with lower muscle mass after adjusting for BMI (35). Nevertheless, the population in their study comprised elderly adults, which may not be generalizable. The current study expanded the population and observed that this relationship also existed in the young population. Skeletal muscle is the largest organ in the body and also the regulator of glucose homeostasis. Insulin could increase glucose uptake by skeletal muscle cells through activation of glucose transporter protein 4 (GLUT4). 80% of postprandial glucose is taken up by muscle from circulation and stored as glycogen (36, 37). Therefore, muscle plays a key role in the association between MUAC and IR.

After adjusting for BMI in the logistic regression analysis, the relationship between TSF thickness and IR disappeared. Further adjustment for WC, which represents visceral fat, indicated that the association between TSF thickness and IR remained uncorrelated. BMI is often used as an indicator of overall obesity. A previous study has revealed that in the Asian population, BMI was weakly correlated with overall fat mass but more strongly correlated with visceral fat mass. As visceral adipose tissue mass increased, IR became more severe (38). This indicated that visceral fat plays a more important role in the development of IR (39). There was a mechanism that may explain the non-significant association between TSF thickness and IR. The amount of ectopic fat accumulation, rather than subcutaneous fat accumulation, has been suggested to be associated with metabolic complications (40, 41). Subcutaneous adipose tissue preferentially stores energy surplus (13, 41). When a cutoff point of subcutaneous adipocyte expansion is reached, it leads to adipocyte hypertrophy, decreased fat synthesis, decreased angiogenesis, and subcutaneous adipose fibrosis, prompting the efflux of free fatty acids and ectopic accumulation, which eventually leads to IR (38, 42). Therefore, the expansion of subcutaneous adipose tissue is an important factor in the development of IR (40, 41).

Additionally, this study explored potential effects on the relationship between mid-arm measurements and IR. Our study found that BMI and WC modification was associated with the negative relationship between MUAC and IR. Although the protective effect of MUAC on IR diminished with the increase of BMI or WC, the relationship between MUAC and IR remained the same among different groups. Previous studies included middle-aged and/or elderly participants, we also included all participant aged above 18 and subjects those who was younger than 40 years old accounted for a quarter of the study population. Age-specific effect was not observed in this relationship, which may suggest that the current findings can also be applied to younger age groups.

This large-scale cross-sectional study has some limitations. First, this study was a cross-sectional study, thus, observing long-term changes in causal relationships was not possible. Second, our study was observational, and residual and unmeasured confounding factors may have existed. Third, only Chinese adults were included in this study, future research exploring whether the results are applicable to other populations is needed.

In conclusion, after adjusting for BMI and WC, MUAC was negatively associated with IR in the Chinese adult population. This relationship was mainly derived from MAMC, while TSF thickness was not significantly associated with IR. Mid-arm measurements can be used as a supplement to BMI to better assess IR. At the same BMI level, a larger MUAC would be protective against IR. The protective effects of MUAC existed not only in elderly, but also in young adults. Our findings may help clinicians to determine IR more accurately and to understand the pathophysiology of IR more clearly in clinical practice.

## Data availability statement

The raw data supporting the conclusions of this article will be made available by the authors, without undue reservation.

## Ethics statement

The studies involving human participants were reviewed and approved by the University of North Carolina at Chapel Hill, the National Institute for Nutrition and Health, and the Chinese Center for Disease Control and Prevention. The patients/participants provided their written informed consent to participate in this study.

## Author contributions

The conception and design of the study: HZ, LH, and JW. Acquisition of the data: JW, LH, NY, and HZ. Analysis and interpretation of the data: JW, LH, and HZ. Draft of the article: JW, LH, HZ, and YL. Critical revision for important intellectual content: JW, LH, NY, LX, FP, WL, HZ and YL. Final approval of the version to be published: All authors. Funding acquisition: HZ and YL. Supervision: HZ and YL. Agreement to be accountable for all aspects of the work: All authors. All authors contributed to the article and approved the submitted version.
